# An Integrative and Modular Framework to Recapitulate Emergent Behavior in Cell Migration

**DOI:** 10.3389/fcell.2020.615759

**Published:** 2020-12-22

**Authors:** Marina B. Cuenca, Lucía Canedo, Carolina Perez-Castro, Hernan E. Grecco

**Affiliations:** ^1^Instituto de Investigación en Biomedicina de Buenos Aires, Consejo Nacional de Investigaciones Científicas y Técnicas (CONICET), Partner Institute of the Max Planck Society, Buenos Aires, Argentina; ^2^Departamento de Física, Facultad de Ciencias Exactas y Naturales (FCEN), Universidad de Buenos Aires and Instituto de Física de Buenos Aires (IFIBA), Consejo Nacional de Investigaciones Científicas y Técnicas (CONICET), Buenos Aires, Argentina; ^3^Department of Systemic Cell Biology, Max Planck Institute for Molecular Physiology, Dortmund, Germany

**Keywords:** fluorescence microscopy, spheroid, cellular automata, migration, image analysis

## Abstract

Cell migration has been a subject of study in a broad variety of biological systems, from morphogenetic events during development to cancer progression. In this work, we describe single-cell movement in a modular framework from which we simulate the collective behavior of glioblastoma cells, the most prevalent and malignant primary brain tumor. We used the U87 cell line, which can be grown as a monolayer or spatially closely packed and organized in 3D structures called spheroids. Our integrative model considers the most relevant mechanisms involved in cell migration: chemotaxis of attractant factor, mechanical interactions and random movement. The effect of each mechanism is integrated into the overall probability of the cells to move in a particular direction, in an automaton-like approach. Our simulations fit and reproduced the emergent behavior of the spheroids in a set of migration assays where single-cell trajectories were tracked. We also predicted the effect of migration inhibition on the colonies from simple experimental characterization of single treated cell tracks. The development of tools that allow complementing molecular knowledge in migratory cell behavior is relevant for understanding essential cellular processes, both physiological (such as organ formation, tissue regeneration among others) and pathological perspectives. Overall, this is a versatile tool that has been proven to predict individual and collective behavior in U87 cells, but that can be applied to a broad variety of scenarios.

## 1. Introduction

Collective cell motion is a complex feature in biological systems, crucial for morphogenetic events, where many single-cell level processes are involved. Aberrations in this coordinated behavior is a hallmark of many pathologies including cancer (Gupta et al., 2011; Stieber et al., 2014; Dirkse et al., 2019; Prager et al., 2019). Chemotaxis, mechanical interactions (with other cells and extracellular matrix) and proliferation have been identified as key mechanisms driving cell migration (Kansal et al., 2000; Khain et al., 2005; Rubenstein and Kaufman, 2008; Charteris and Khain, 2014; Li et al., 2017; Manini et al., 2018). How these processes individually contribute to the emergent behavior is not fully understood as we are limited to either observing single cells or the collective behavior at the multicellular level.

Mathematical models are useful in delineating the role and influence of these individual processes, otherwise experimentally inaccessible. Early studies tackle single-cell movement as a random walker (Fuert, 1920), but this description does not recapitulate the behavior if cell colonies are analyzed or microenvironmental conditions are considered. More complex mathematical frameworks have been developed in continuous models using differential equations. Although such models are computationally inexpensive, the output is a density function that does not reflect single-cell behavior and thus fails to predict subpopulations and patterns in cell colonies (Hatzikirou et al., 2005). On the other hand, in cellular automaton discrete models cells move according to specific rules or probabilities that depend on the neighboring distribution. This approach would allow us to describe the social behavior within cell communities at the single-cell level. However, the rules that drive the automatons are static and do not consider intracellular and molecular mechanisms (Tanaka et al., 2009). To the best of our knowledge, there are no existing models that integrate the broad diversity of biological mechanisms needed to fully predict cell migration.

We developed a simple, fast, powerful and discrete two-dimensional approach that accurately predicts cell migration by considering random movement, proliferation, chemotaxis and mechanical interactions. The algorithm allows for the specification of the initial cell number and colony geometry, as well as the active mechanisms in play. The modular construction of the algorithm allows the user to tune every single aspect of the mechanisms, and make predictions of complex cell arrangements from single-cell characterization. This makes our algorithm a powerful tool that can be adapted to simulate a variety of other complex processes. Wound healing, cell invasion, and morphogenetic events can be addressed even in systems lacking spheroid formation.

## 2. Results

### 2.1. Single-Cell Behavior Gives Insights Into Colony-Scale Observations

**Figure 1:**
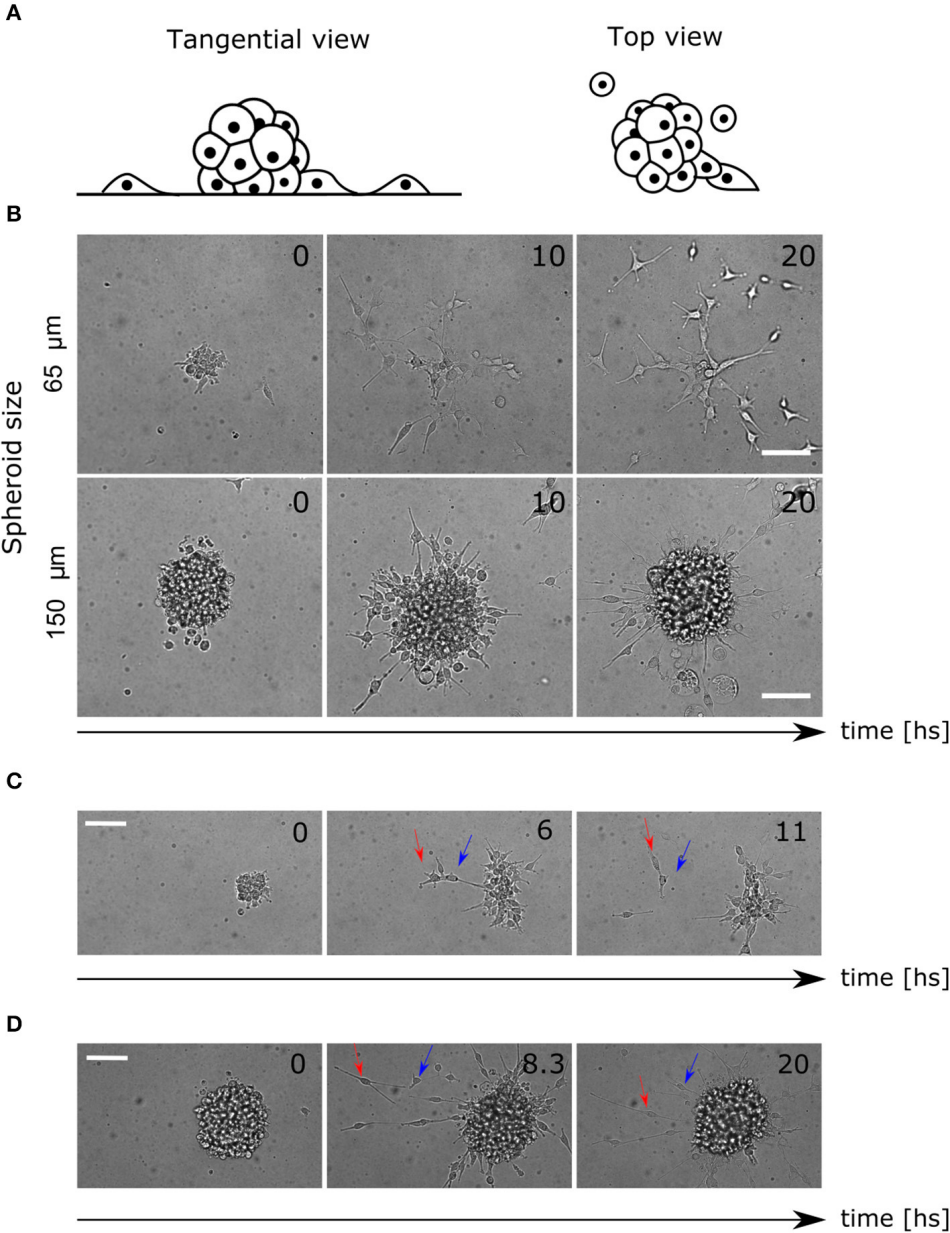
Colony and single cell-level migratory behavior. **(A)** Lateral and top view diagram of cell migration away from the spheroids. The cells are constrained by the Geltrex surface to migrate in 2D trajectories. **(B)** Time-lapse of bright field images where different migration patterns are shown for small and large spheroids. **(C)** Time-lapse of spheroid migration where mechanical interactions between cells are evident. The leading cell (red arrow) migrates away from the colony pulling the second cell (blue arrow). **(D)** Time-lapse of directed radial behavior of two cells (arrows). In the first frame (8.3 hs) they move away and finally (20 hs) they return to the colony again. Scale bar 100 μ*m*.

Glioblastoma (GBM) U87 cells spheroids expressing the nuclei marker pBABE-H2BGFP, placed in Geltrex coated multiwells and covered with fresh stem medium, were imaged for 24 h (Figure 1A, see Material and Methods sections 4.1 and 4.2). Different profiles have been observed within the range of spheroid diameters used (60–200 μm). While in smaller colonies most of the cells detached and migrated, in larger colonies they remained clustered as reported previously (Figure 1B, Supplementary Videos 1, 2) (Puliafito et al., 2015).

Furthermore, we saw an elongated morphology in cells performing migration, interacting with each other, producing collective migration in a radial protrusion away from the colony. Other cells did not show protrusions but migrated in pairs, which reinforced the existence of mechanical interactions (Figure 1C, Supplementary Video 3). Finally, some other cells migrated as single cells randomly for the first hours, but then, radial movement and re-clustering were observed (Figure 1D, Supplementary Video 4).

Taking together these observations, we proposed that random movement was not enough to explain the invasive profile of the colonies. We hypothesized that short-range mechanical interactions between cells might affect motility, and another long-range chemotactic process would radially affect migration direction. Previous studies consider chemotaxis of glucose as an attractant factor driving cell migration in GBM (Khain et al., 2005; Bao et al., 2019). We hypothesized that the cells are dominated by random movement and mechanical interactions during the early hours of the experiment. Later, the chemical diffusion and concentration of a chemo-attractant factor self-generated by the cells would drive re-clustering. This could explain why in large spheroids there are fewer migrating cells, considering that the concentration of chemo-attractant is higher.

Notably, thanks to the nuclei marker visualization, few mitosis events were observed in our time-lapses. We decided to explore within the simulations the influence of proliferation. To keep generality, is a mechanism that cannot be discarded in other cell lines, longer duration of the experiments or higher cell number. Therefore, we added it as a fourth mechanism during cell migration.

### 2.2. Quantification of Cellular Motility Reveals Time and Colony Size Dependent Behavior

**Figure 2:**
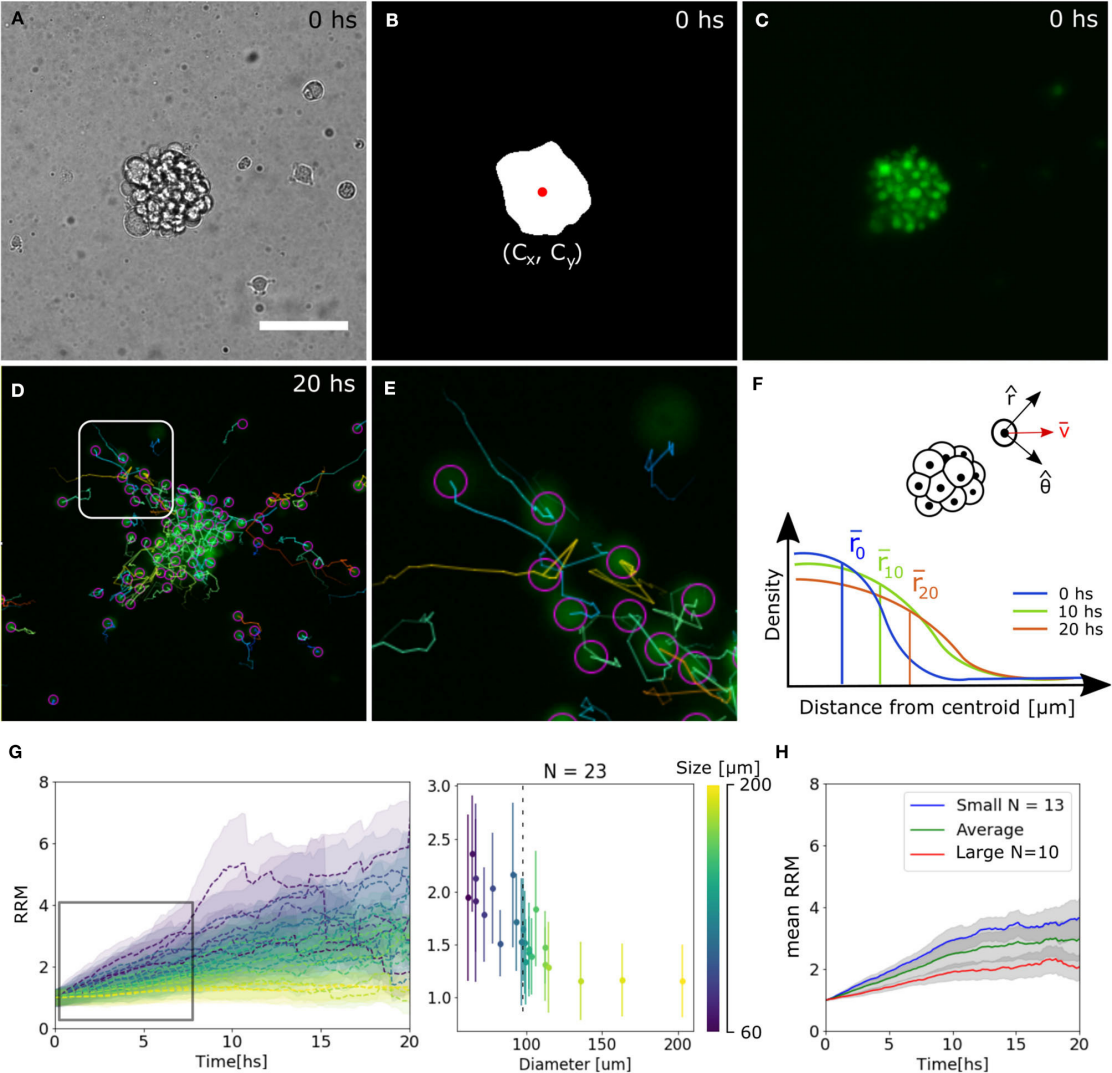
Tracking analysis of individual nuclei. **(A–C)** Bright field, binary mask with centroid and fluorescence channel of spheroid at time 0. **(D,E)** Individual cell tracks and close-up. The track for the last 10 time-points is indicated by a straight line and the final nuclei position with a circle. **(F)** Diagram of polar coordinates calculated from the centroid of the spheroid, distribution of radial distances and mean value. **(G)** Experimental curves of mean RRM for different sizes of spheroids (left) and RRM after 7.5 hs of migration vs. diameter (right). We can define a threshold around 100 μm (dotted line) where the behavior is constant for larger spheroids. **(H)** Average mean RRM for small, large and all sizes of spheroids.

To extract quantitative results that validate our observations, bright field images were segmented to identify the centroid of the spheroid. Then, the trajectories of single-cells expressing the nuclei marker pBABE-H2BGFP were obtained, and for each spheroid, the mean relative radial migration (RRM) was calculated at every time-point (Figures 2A–F, see Materials and Methods section 4.3). We chose a nuclear marker because its morphological structure is stable throughout the cell cycle and cellular migration (Cliffe et al., 2017). The mean RRM is a measure of the distribution of cells around the centroid of the spheroid. Thus, a spheroid that remained clustered has a mean RRM of 1, while a spheroid whose cells doubled mean distribution has a mean RRM value of 2.

We observed that for larger spheroids the mean RRM is close to 1, while smaller spheroids could migrate six times their mean initial radius. Analyzing the dynamics after 7.5 h of migration (region of linear increase in RRM), we defined a threshold at 100 μm separating a constant and divergent behavior for large and small spheroids, respectively. From now on, we will show the average data on each side of the threshold (Figures 2G,H).

### 2.3. Single-Cell Model Accurately Predicts Emergent Colony Behavior

In our model, a single isolated cell in a substrate can migrate with a random component, where all directions are equally likely and a diffusion coefficient describes the area covered per unit of time (Fuert, 1920). To estimate this parameter, we analyzed the movement of single cells in a low-density monolayer in the same culture conditions as the spheroids during the migration assays (see Material and Methods section 4.2). Having single cells apart from each other, it is possible to quantify the random motion only without cells interacting mechanically with each other and having a non-significant concentration of self-generated chemoattractant. We obtained a diffusion coefficient of *D*_*cell*_ = (0.21±0.04) μ*m*^2^/*s* (mean ± SEM, see Supplementary Figure 1).

**Figure 3:**
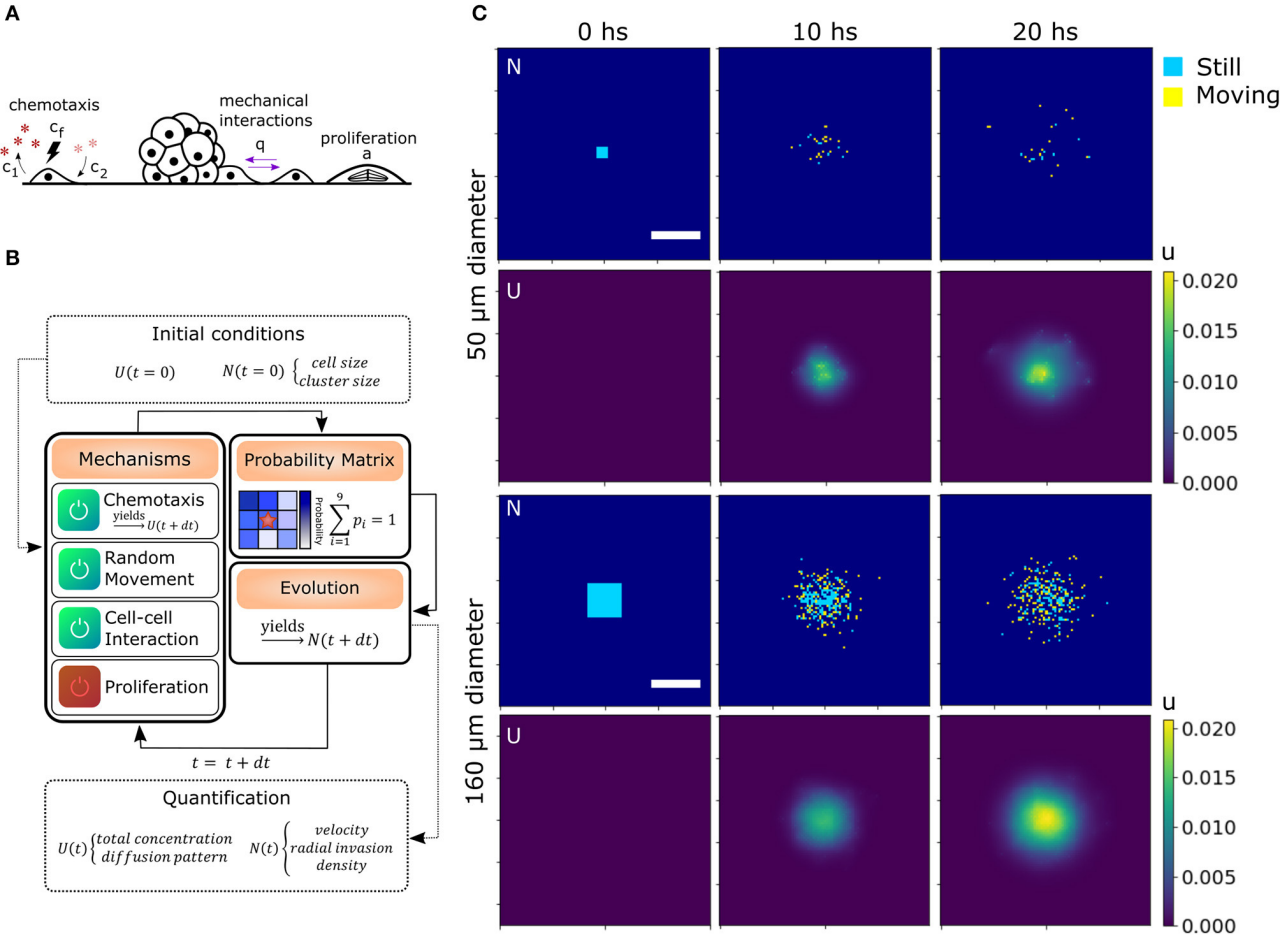
Algorithm architecture and operation. **(A)** Additional mechanisms considered from experimental observations: Chemotaxis, mechanical interaction between cells and proliferation. **(B)** Simulation steps: Initial conditions for U and N are stated. The user select the active mechanisms and its parameters. The time iterations are performed considering the configuration, obtaining the probability matrix for each cell and simulating the evolution in a differential time step. Then, the process starts again for as many iterations as needed. Finally, morphological parameters are extracted from each time point. **(C)** Matrices of cell and chemical distribution (N and U) at times 0, 10, and 20 h for a small 50 μm (top) and big 160 μm (bottom) geometry. Cells in yellow moved in the last iteration while cells in blue remained still. Scale bar = 200 μm.

Mechanical interactions were included in the model defining a probability *q* for two cells to interact with each other at first (Charteris and Khain, 2014) and second neighbors (can be extended to further neighbors, see section 4.4.3). Chemotaxis was modeled by assuming an effective chemo-attractant produced and consumed by cells with rates *c*_1_ and *c*_2_, respectively (Keller and Segel, 1971; Hillen and Painter, 2008). It diffuses with a rate *D* = *D*_*chem*_/*D*_*cell*_ times faster than the cells effectively providing a mid-range cell-to-cell interaction. This quantity is delimited by the stability conditions of the chemotaxis equation (see Materials and methods, section 4.4.2). When this chemical gets to the membrane of neighboring cells, some molecules attach to specific receptors and unchain a signaling pathway to produce migration in the direction of the chemical gradient (Kim et al., 2009). The strength of the chemo-attractant to drive cells in a particular direction is given by the quantity *c*_*f*_. Finally, we also defined α which is the probability of one cell to undergo division during the time step defined previously. As we discussed before, there are only a few divisions in our migration assays, thus α = 0 unless stated otherwise. All the mechanisms considered in addition to random movement are illustrated in Figure 3A.

To computationally run the model, we used a discrete lattice (matrix) to describe the cell (*N*) and chemical (*U*) distribution. Defining the cell size and time step is sufficient to set the diffusion coefficient *D*_*cell*_ in *N*. Each cell is a square of area 100 μ*m*^2^ (average size of 10 μ*m*). Then, in our simulations, we defined a time step of 7 min (205 iterations in total for 24 hs *in silico* experiment). We also defined a probability *r* for the cells to perform random movement. In this way, we were able to alter *D*_*cell*_ in our simulation without changing the geometry of the cells and the number of iterations, which would require to re-scale the other parameters of the model. This might be of interest as we will discuss further ahead. In our simulations, *r* = 1 unless stated otherwise.

To perform the simulations, we used a modular approach to combine all the biological processes, translate them into probabilities, and then, consider each cell as an automaton. Cell occupancy *N* and chemo-attractant distribution *U* was iteratively evolved according to the described equations (see sections 4.4.2 and 4.4.5). These probabilities change in each iteration (time-point) based on the diffusion of the chemical and cell distribution. The initial conditions, such as cluster (spheroid) size and geometry *N*(*t* = 0), and chemical concentration *U*(*t* = 0), are given by the user (Figures 3B,C). To delimit the possible values of each parameter, we compared the RRM results of the simulations with the random movement *in silico* and the experimental results. Only the parameters with RRMs between the experimental and random curves are candidates (see Supplementary Figure 2).

**Figure 4:**
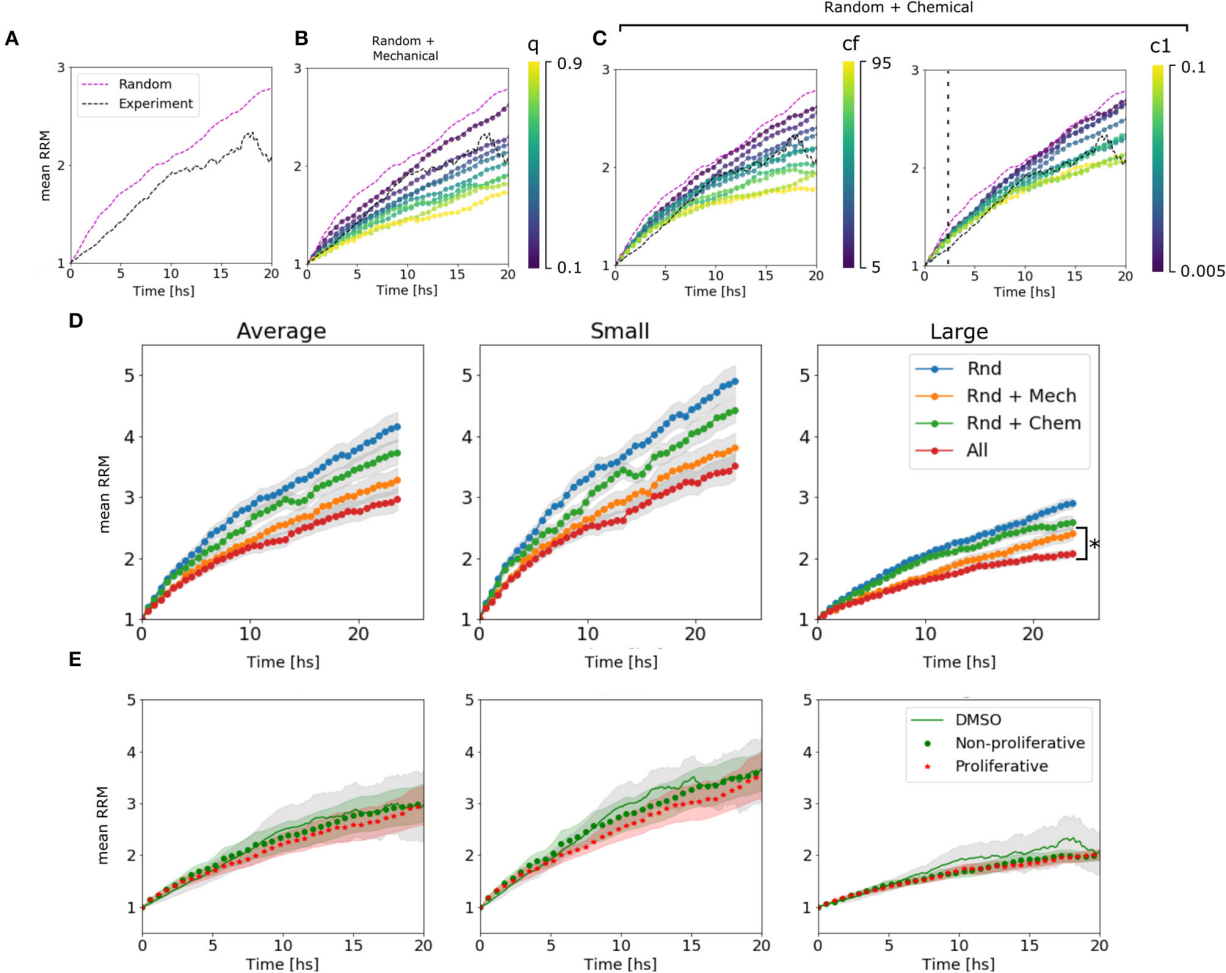
Algorithm behavior and fitting procedure. **(A)** Dynamic range available for the estimation of parameters in the average of large spheroids. We are confined to the region between the random movement simulated curve and the experimental curve. **(B)** Simulated curves for different values of *q* change their slot. We see that we can move in the range from 0 to 0.3. **(C)** Simulated curves for different values of *c*_*f*_ change the asymptotic value. The range between 5 and 45 is valid. **(D)** Simulated curves for different *c*_1_ change the slot above ~2 h of migration. All the explored range is, in principle, valid. **(D)** Change in RRM due to the presence/absence of different mechanisms in the average of all, small and large spheroids: In blue, only random movement was taking place; In orange, mechanic interactions were added; In green, it is shown the effect of random movement plus chemotaxis. Finally, all the mechanisms together in red. We can see that the difference between adding chemotactic effects is not significant for small spheroids but remains important for large spheroids (*p-value < 0.05, *N* = 24 spheroids). **(E)** Comparison between experimental curve and simulated curves with and without proliferation.

We could assign different behaviors of the curves to different parameters. The mechanical parameter *q* is strongly associated with the slope or velocity of migration, which makes sense in a scenario where the cells at *t* = 0 are cluster together and the mechanical interactions have a higher impact. While *c*_1_ is associated with the velocity only after ~2 h, once the chemical has accumulated. On the other hand, *c*_*f*_ changes the final value of the mean RRM (Figures 4A–C and Supplementary Figure 3). The diffusion coefficient *D* showed slight differences in the emergent behavior only in large spheroids, where the chemical concentration is higher. In this scenario more diffusion means a lower chemical gradient in the spheroid, and thus, the cells can arrange in a wider distribution with higher mean RRM (Supplementary Figure 2). This value of *D* = 50 returned a *D*_*chem*_ = (630 ± 120) μ*m*^2^/*min* which encloses, for example, the diffusion coefficient of glucose in water (*D*_*gluc*_ = 670–700 μ*m*^2^/*min*) (Øyaas et al., 1995; Andriesse and Hollestelle, 2001). This is not enough to conclude which is/are the molecule(s) involved in the chemotaxis, given the fact that there are probably attractants and repellents. In our model, we are considering an effective chemotaxis that showed to be positive (attractant).

To determine the influence of each mechanism in the simulated results, we turn off one mechanism at a time in the model. Then, we compared the final mean RRM values with the random movement and the complete model simulated curve. As hypothesized, the chemotaxis effect had a significant influence on large spheroids, while it did not change the global behavior on small clusters (see Figure 4D). This indicates that the complete model is not only the sum of its components and that the mechanisms at the single-cell level return emergent responses in different size-scales. We also test whether the proliferation affects cell migration pattern setting the probability α to 0.0007–0.0012 [joint probability between the doubling rate of 19–30 h (Charteris and Khain, 2014; Rodríguez-Lozano et al., 2019) and 20% of proliferative cells within the spheroid (Aaberg-Jessen et al., 2013)], concluding that there is no significant difference within 24 h (Figure 4E, see Material and Methods section 4.4.4).

**Figure 5:**
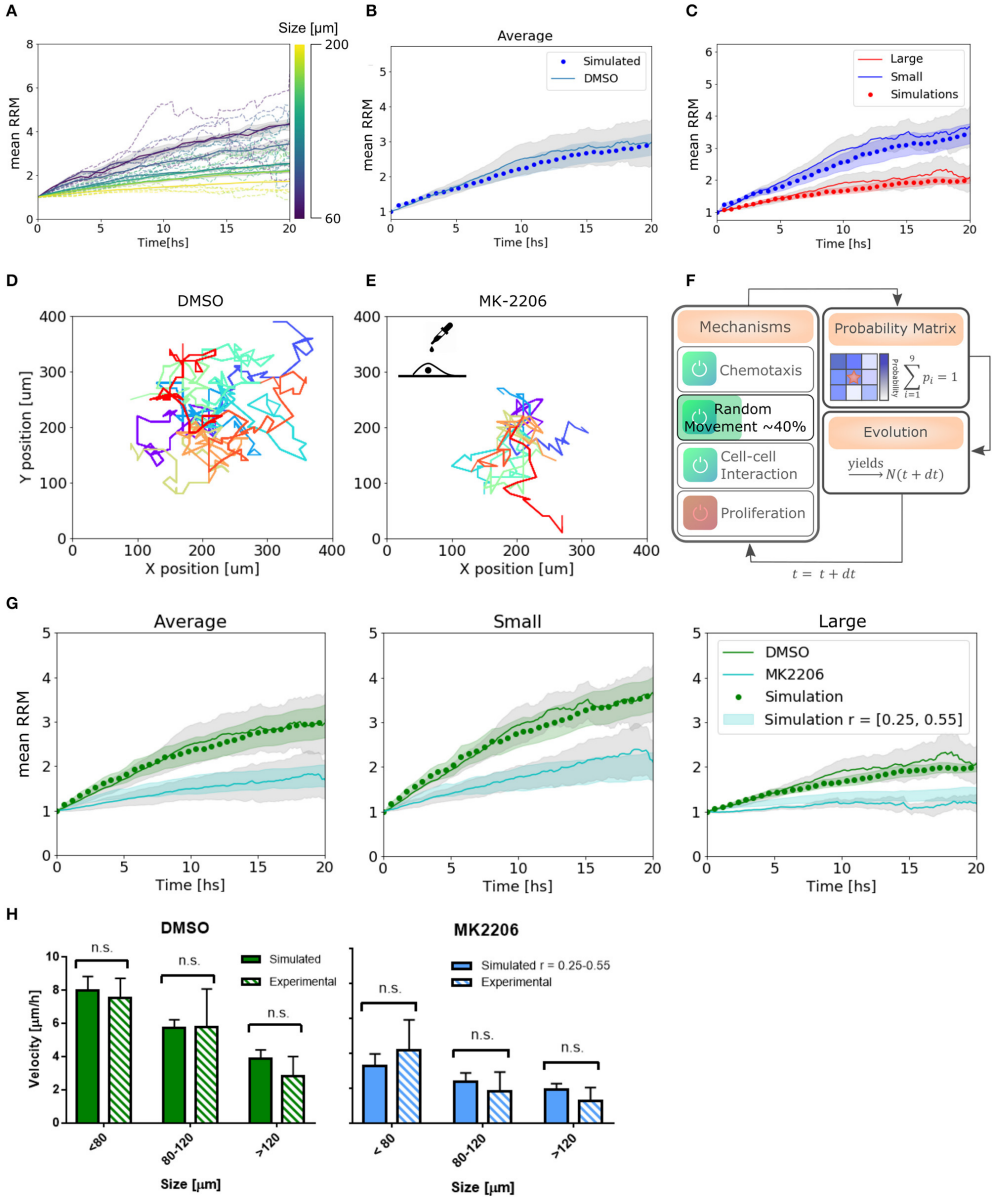
Simulations reproduce migratory behavior and predict drug-assay results. **(A)** Simulated curves of mean relative radial migration for different size of spheroids. **(B)** Average mean RRM for all sizes. Experimental and simulated curve. **(C)** Average mean RRM for small and large spheroids. Experimental and simulated curves. **(D,E)** Single cell trajectories for the diffusion coefficients measured for DMSO and MK-2206 treated cells in monolayer. **(F)** Modification in the model: Only random movement (diffusion) of the cells was altered in the same range indicated by experimental results. **(G)** Prediction of the model and experimental results with MK-2206 treatment for all spheroids (left). Prediction and experimental results for small (*N* = 12) and large (*N* = 11) spheroids separately. **(H)** Average invasion velocity calculated from linear fit of RRM data for each spheroid during the first 7.5 h of invasion. Non-significant differences have been observed for simulated and experimental data both for MK-2206 treatment and DMSO control. While for DMSO the simulated data corresponds to the optimum set of parameters, for MK-2206 an uniform distribution of the random parameter *r* between 0.25 and 0.55 was used.

Finally, we found a set of parameters that not only fit the experimental results in average, but also replicate the behavior in single spheroids and on each subset of small and large colonies: *D* = 50, *c*_1_ = 0.035, *c*_2_ = *c*_1_/2, *c*_*f*_ = 20, and *q* = 0.3 (Figures 5A–C). The algorithm performed each simulation (total number of spheroids = 24) in 10 min (see Materials and Methods section 4.4).

### 2.4. Experimental Perturbation on Cellular Mobility Impacts the Emergent Behavior, Following the Algorithm Prediction

To test the capability of the model to predict other cell behavior, we created a perturbation in the random motion mechanism experimentally. Single cells in a low-density monolayer were treated with AKT1 allosteric inhibitor MK-2206 (Narayan et al., 2017). Akt is a major signaling molecule that is highly expressed and aberrantly activated in GBM, contributing to the malignant phenotype (Nakada et al., 2013). It is often used in combination with Notch inhibitors and has been proved to be a migration inhibitor, without altering proliferation, in a dose-dependent manner (Jin et al., 2013; Djuzenova et al., 2019).

The diffusion coefficient was quantified from single-cell trajectories as described previously being just 40 ± 15% (mean ± SEM) of the value of cells treated with the vehicle DMSO (Figures 5D,E, see Supplementary Figure 1). Thus, instead of changing the time step of the iterations to modify *D*_*cell*_, which would have changed also all the set of parameters found, we simply changed the probability to perform random movement. We simulated a set of spheroids *in silico* with *r* between 0.25 and 0.55 to obtain an effective *D*_*cell*_ to match the experimental measurements (Figure 5F). Only then, we treated the spheroids with the migration inhibitor and proceeded with the analysis as usual. Modifying only the parameters associated with the altered mechanism, we observe a good agreement between the model predictions and experimental results, both for the average and subsets of data (Figure 5G). Moreover, we calculated the mean velocity in the first 7.5 hs of simulated spheroid invasion (linear regime) and obtained non-significant differences compared to the experimental results for three different size subsets (Figure 5H). This indicates that not only our model can fit our experimental data correctly, but also predicts dynamic and average quantitative parameters related to collective cell migration.

## 3. Discussion

The tumor cell migration comprising cell random movement, mechanical interactions, and chemotaxis is extremely complex to interpret. Our data from single-cell and colony behavior analysis allowed us to develop a concise and versatile tool that contributes toward understanding tumor-associated biological processes. Our approach is simple and fast, and can be extended far beyond the GBM model and adapted to other cell lines and scenarios including migration, proliferation, morphogenesis, and wound healing. We have shown how, from a simple determination of the cell diffusion coefficient, our model can replicate a set of migration assays of U87 cellular spheroids. The only experimental requirement would be a nucleus labeling, either by staining or retroviral transfection, to determine each cell position.

We demonstrated the relevance of all the mechanisms proposed in the model and the influence in the migratory process. We found that while random movement and mechanical interactions have a high impact on the mean RRM, they are not sufficient to explain the invasive pattern in large spheroids (>100 μm) where chemotaxis becomes significant. Adjusting random movement of the cells, we were able to make good predictions on the effects of the migration inhibitor MK-2206, obtaining a good agreement between the *in silico* and *in vitro* cell behavior.

No considerations regarding the mechanical properties of the environment have been made. Our model assumes that the rigidity of the substrate is homogeneous and affects the velocity at the single-cell level. Therefore, it is adequate to determine experimentally the diffusion coefficient of isolated cells to encode the mechanical properties. However, the heterogeneous composition of the “real” tumor microenvironment could modify (or affect) the diffusion coefficient of individual cells and alter the emerging behavior of the colonies given by cellular interactions with the surroundings. Since current cancer treatments do not address the dynamic regulation of the tumor microenvironment, primarily responsible for tumor progression and resistance to treatment; future adaptations to this first base model allowing for the presence of microglia and different tumor cell identity states (proliferative, migrating, senescent, etc.) (Tektonidis et al., 2011; Friedmann-Morvinski, 2014) should be considered to evaluate and predict with greater fidelity the cellular behavior *in vivo*.

The traditional therapeutic approach for GBM is surgical resection, where most of the tumor mass is removed. However, some residual cells mostly at the invading edge could persist after treatment (i.e., resection plus chemo and radiation therapy) and have been hypothesized to be responsible for the recurrences observed in patients (Swanson et al., 2000; Stupp et al., 2005). Tumor stem-like cells have been identified as one of the main subpopulation responsible for the tumor recurrence, and though some gene markers and molecular pathways have been recently identified (Wang et al., 2016; Minata et al., 2019), the area is still under investigation. Our finding could contribute to understanding how the different mechanisms considered impact at different colony-size scales and regulate the migratory behavior of tumors. Our results could also be used to integrate with new findings in the area of research and explore the impact of new biomarkers and test future therapeutic interventions.

The cell line used in this study (U87), has been chosen as a model of GBM in over 2000 publications since they reproduce the oncogenic cell signaling in the original tumor (Lenting et al., 2017). Future studies using other GBM and tumor cells (commercial lines, and cells derived from patients), will be necessary to expand the generality of our observations. The use of these cells will also be useful to better understand the impact and scope of the model, as well as the possibility of proposing new questions, given that patient-derived cells better represent tumor biology and the heterogeneity found daily in the clinic.

The results and the model characterized in two dimensions from spheres culture here are the first approaches to extract the biological parameters, to validate the mechanisms proposed, and to obtain predictions of drug assay experiments *in vitro*. Scaling into the third dimension is an important upcoming step to further predict invasive behavior *in vivo*. Therefore, our contribution will be relevant in the field in order to move forward to try to understand the complex tumor migration and cancer.

## 4. Materials and Methods

The steps involved in producing the experimental and corresponding simulated results were performed in the following order: (i) migration assay experiments with GBM spheroids, (ii) image analysis and data processing, (iii) selection of the biological actuators, (iv) identification of mathematical analogs for the simulations, (v) experimental and *in silico* determination of parameters, (vi) prediction of drug assay response, and (vii) test the predictions experimentally.

### 4.1. Cell Culture and Viral Infection

Cell line U87MG was acquired from ATCC, kept frozen immediately after receipt or used in culture <2 months and routinely tested negative for mycoplasma. ATCC cells are characterized by Short Tandem Repeat (STR) profiling (Ferreyra-Solari et al., 2016). U87 cells were cultured in DMEM supplemented with 10% fetal bovine serum, Penicillin/Streptomycin, and L-Glutamine to express several of GBM markers as determined previously (Lenting et al., 2017). Cells were kept at 37°C under 5% CO2 humidified air. The plasmid pBABE-H2BGFP was a gift from Fred Dick (Addgene plasmid # 26790; http://n2t.net/addgene:26790; RRMD:Addgene_26790). Retrovirus was created using standard protocols and introduced into U87 cells. Briefly, viruses were harvested at 48 and 72 h, cleared of cell debris. To establish the stable U87-H2GFP cell line, monolayers were subjected to 2 rounds of infection. After retroviral transduction, GFP-positive cells were sorted by FACS, collected for amplification and maintained with puromycin (1 μ*g*/*ml*). For neurosphere induction, U87 GBM cells were grown to 90% confluence, trypsinized, and plated in ultra-low adhesion multi-well plates (Corning) in neural stem cell (NSC) medium or DMEM F12 supplemented with B27, N2, 20 ng/ml bFGF, 20 ng/ml EGF, 2 mM L-glutamine, 2 mM non-essential amino acids, 50 U/ml penicillin/streptomycin (Sigma, USA). After 5 days, the number of spheroids was quantified using 10× magnifications under a phase contrast microscope (Carl-Zeiss, AxioObserverZ1), an AxioCam(HRm) camera (Carl-Zeiss, Germany) and Zen pro2011. It is considered a spheroid, a cell cluster bigger than 40 μ*m* in diameter (Sart et al., 2017; Bodgi et al., 2019).

### 4.2. Migration Assays, Migration Inhibition, and Microscopy

Twelve well plates were coated with 400 μl of LDEV-Free Reduced Growth Factor Geltrex (Invitrogen), adding 1 ml of serum-free fresh medium with spheroids in suspension on top. After half an hour, spheroids were attached to the coating. Bright field images were taken every 10 min for 24 h in a Zeiss AxioObserverZ1 inverted microscope with Live Imaging System.

To inhibit migration the process was the same as described above, but Akt1 inhibitor MK-2206 (Cayman Chemical #11593) was added to the fresh medium. A concentration of 7 μM was used to inhibit migration, without altering proliferation and cell viability as described before (Jin et al., 2013). Control experiments were performed with the vehicle, DMSO in this case. To determine cell's diffusion coefficient a low-density monolayer was cultured in the same conditions as the spheroids, with MK-2206 and the vehicle.

Registration in all cases was performed using 10× magnification under a phase contrast microscope (Carl-Zeiss, AxioObserverZ1) with Live Imaging system, an AxioCam(HRm) camera (Carl-Zeiss, Germany) and Zen pro2011. Bright field and fluorescent images were obtained every 10 min for 24 h.

### 4.3. Image and Statistical Analysis

Analysis of time-lapses was performed in a custom built Python pipeline and Fiji (Schindelin et al., 2012). First, a binary segmentation based on Otsu's criteria was performed after a Gaussian filtering using the Scikit-Image library (van der Walt et al., 2014) on the bright field images. Morphological parameters, such as the centroid and diameter were extracted from these binary images. Fluorescent nuclei positions were tracked using the Fiji plugin TrackMate (Tinevez et al., 2017). The radial position from the centroid was quantified for each cell in the spheroid, averaged and normalized to obtain the mean relative radial migration

$$\begin{array}{l} meanRRM\left(t_{j}\right)=\frac{{\langle \sqrt{{\left(x_{i}\left(t_{j}\right)-x_{c}\right)}^{2}+{\left(y_{i}\left(t_{j}\right)-y_{c}\right)}^{2}}\rangle }_{i}}{{\langle \sqrt{{\left(x_{i}\left(t_{0}\right)-x_{c}\right)}^{2}+{\left(y_{i}\left(t_{0}\right)-y_{c}\right)}^{2}}\rangle }_{i}}, \end{array}$$

where *x*_*i*_(*t*), *y*_*i*_(*t*) is the position of the *i*th-cell at each time-point and *x*_*c*_, *y*_*c*_ is the centroid of the spheroid. Note that the average is over the number of cells and not over time.

In single cells, the diffusion coefficient was calculated from single trajectories as

$$\begin{array}{l} d_{cell}=\frac{1}{4T}\overset{N}{\underset{j=1}{\sum }}\left[{\left(x\left(t_{j}\right)-x\left(t_{j-1}\right)\right)}^{2}+{\left(y\left(t_{j}\right)-y\left(t_{j-1}\right)\right)}^{2}\right], \end{array}$$

where *T* is the total duration of the trajectory and *N* is the total amount of time-points. Then, *d*_*cell*_ values for all cells were averaged to obtain *D*_*Cell*_. To analyze the data, Pandas package was used (McKinney, 2010). The statistical test used was the non-parametric Kolmogorov-Smirnov analysis to compare cumulative distributions.

### 4.4. Mathematical Modeling

A discrete approach was used to simulate the cell clusters. Basically, a squared lattice, represented by a matrix *N*, was filled with zeros (for empty spaces) and ones (for occupied spaces). The squared lattice has cells of the same size than the actual U87 cells average size, in this case, 10 μm. Consistently, dimensionless quantities were only considered in the matrix. Then, *N* is a *m* × *m* matrix, being *m* an appropriate size for the cells to migrate without reaching the borders. In this case, *m* = 200 (which is equal to 2,000 μm) was considered enough. The initial condition for *N*(*t* = 0) is a *d* × *d* cluster (*d* << *m*) of one values centered in the matrix, surrounded by zero values. The value of *d* will vary from 5 to 15 approximately (50–150 μm), which is the range of the real diameters of the spheroids. The approach is two-dimensional in the vertical and horizontal axes (*y* and *x*, respectively), represented by *i* and *j* matrix indexes (rows and columns).

The evolution of this matrix will be computed based on four different biological actuators considered of relevance after analyzing the experimental results. The first one is random movement or Brownian motion of the cells. The second one is the chemotaxis generated by some chemo-attractant/repellent factor segregated by the cells. Finally, mechanical interaction between the cells will be considered. With these three processes, a probability matrix *P* ∈ ℜ^3×3^ will be calculated for each cell in the grid. That matrix will represent the probability of the cell to remain in the same place (*P*_[2,2]_) or moving to a neighboring space. After selecting the direction of movement, the cell will move toward that space only if it is free, and will remain in the same space if it is occupied. Cells can also proliferate with a given probability α, and in that case, they will not move. After obtaining the new matrix *N*(*t* = *dt*), the process starts again (see Figure 4B).

The time step *dt* should reflect the time in which one cell is able to move its own diameter. In other words, it is linked with its diffusion coefficient directly. In this case, it was determined to be ~7 min. To move forward a dimensionless quantity, we defined the total unity of time as 24 h. Then, *dt* = 7*min*/24*h* ~ 0.0048. We could have considered a unity of time of 12 h, but it would have an influence on the stability of the chemotaxis equation (see section 4.4.2) restricting the diffusion constant *D* and it would have doubled the computing time.

Using a processor Intel(R) Core(TM) i7-6500U and 8 GB of RAM, reproducing a complete experiment (a total of 24 spheroids of different sizes) took 10 min.

#### 4.4.1. Random Movement

The random component of the probability matrix for all the cells will be a Gaussian kernel *G* ∈ ℜ^3×3^ with standard deviation σ = 2 spaces. The central value of this matrix *G*_[2,2]_ was set to zero, so the cell is enhanced to move away, and the rest is re-normalized so

$$\underset{k,l}{\sum }G_{\left[k,l\right]}=1.$$

#### 4.4.2. Chemotaxis

The movement of a single cell toward the direction of a chemical gradient is called chemotaxis. If the chemical is an attractant, the cell will move toward the positive gradient. But, if it is a repellent factor, the cell will move toward the negative gradient. Based on the approach used in Charteris and Khain (2014), we also describe the chemical concentration in a discrete squared lattice, represented by the matrix *U*. The evolution of the chemical concentration in two dimensions is ruled by the differential equation

$$\begin{array}{l} \frac{\partial u}{\partial t}=D\frac{\partial^{2}u}{\partial x^{2}}+D\frac{\partial^{2}u}{\partial y^{2}}+c_{1}n-c_{2}nu, \end{array}$$

where *u* and *n* are continuous variables describing the chemical and cell concentration, *D* = *D*_*chem*_/*D*_*cell*_ is the dimensionless diffusion coefficient, *c*_1_ is the generation constant, and *c*_2_ is the degradation constant. In this case the production term is proportional to the concentration of cells, because the chemical is being produced by the cells, and the degradation term is proportional to the concentration of the chemical.

The initial condition is *u* = 0 in all the space, and the way to proceed is to solve the differential equation using the initial cell concentration to find *u*(*t* = *dt*). But we have a discrete *N* so, in order to solve the equation, we performed finite differences to find the discrete chemical concentration *U*, a matrix with the same size as *N*. At the end, we obtained

$$\begin{array}{l} U\left[t+dt\right]=MU\left[t\right]+U\left[t\right]M+c_{1}dtN\left[t\right]-c_{2}dtU\left[t\right]*N\left[t\right] \end{array}$$

where *dt* is the discrete time.

*M* is a tridiagonal matrix given by

$$M=\left(\begin{array}{ccccc} \frac{1}{2}\Delta & \lambda & 0 & \ldots & 0\\ -\lambda & \frac{1}{2}\Delta & \lambda & \ldots & 0\\ \vdots & \vdots & \vdots & \ddots & \vdots \\ 0 & 0 & 0 & \ldots & \frac{1}{2}\Delta \end{array}\right),$$

where Δ = 1 − 4λ, λ = *Ddt*/*h*^2^ and *h* is the discrete step in length (*h* = 1, in this case). The condition for the stability of the solutions is *dt* ≤ *h*^2^/2*D*.

In each iteration, the cell will sense the neighboring chemical concentration in both directions and compute the weighted gradient

$$\mu_{x}=\frac{U_{\left[i,j+1\right]}-U_{\left[i,j-1\right]}}{{\left(1+3u\right)}^{2}}$$

$$\mu_{y}=\frac{U_{\left[i+1,j\right]}-U_{\left[i-1,j\right]}}{{\left(1+3u\right)}^{2}}.$$

The equation reflects how the chemotaxis effect is lower for higher concentrations of the chemical, which produces a saturation of the cell membrane receptors. Also, we can see how the effect is larger for higher gradients.

In the end, the probability of moving toward the gradient is given by |μ|. And thus, the probability matrix *C* will be filled with zero values except for the index with maximum gradient modulus.

#### 4.4.3. Mechanical Interaction

Based on observations of the collective behavior of the cells in the spheroids, a mechanical interaction between cells was added. In this case, cells can interact with the first neighbors by performing a convolution between *N* and a 3 × 3 matrix *O* filled with ones. The size of the matrix filled with ones should be changed in order to consider further neighbor interactions. After convolving *N* and *O*, we cut the matrix in small 3 × 3 matrices around each occupied space. To avoid the simulated cells to interact with themselves, we subtract the matrix *O* and normalize to obtain *I* ∈ ℜ^3×3^, a map of forces at first neighbors.

#### 4.4.4. Proliferation

A probability per unit of time α can be assigned to consider proliferation. In this case, for each iteration and cell, the algorithm will decide whether if proliferation will take place or the cell is going to move. If it chooses to proliferate, then a free space must be available. The mother cell will remain still and a daughter cell will be assigned randomly to one of the free neighboring spaces. The proliferation probability is associated with the life cycle (~19–30 h). Then, the probability per unit of time, considering our time step, is the ratio step/cycle ~0.004–0.006.

In our scenario, not all the cells are proliferative. Only around 15–20 % of the cells will divide. Then we have to calculate the joint probability of the cells to be proliferative and undergo division per unit of time. In other words, we had to calculate

$$\begin{array}{l} \alpha =P\left(A\cap B\right)=P\left(A|B\right)P\left(B\right) \end{array}$$

where A represents the process of dividing and B represents a proliferative cell. Then, considering *P*(*A*|*B*) = 0.004 − 0.006 and *P*(*B*) = 0.15 − 0.2, we obtained α = 0.0007 − 0.001.

#### 4.4.5. Complete Model

All the interactions and processes described above can be switched on and off. When they act at the same time, the combined probability matrix for each cell is given by *P*′ = *r* * *G* + *c*_*f*_ * *C* + *q* * *I*. The parameters *r* and *q* are numbers between 0 and 1 indicating the proportion of influence of the random motion to the total probabilities and strength of mechanical interactions between cells. While *c*_*f*_ is the chemotaxis coefficient, that indicates the nature (*c*_*f*_ < 0 if repellent, > 0 if attractant) and strength of the chemical. In all the simulations *r* = 1 unless specified otherwise. Finally, *P*′ is normalized to obtain *P*. Be aware that if only random movement is taking place, the last normalization is not needed.

## Data Availability Statement

The raw data supporting the conclusions of this article will be made available by the authors, without undue reservation. Code is available at: https://github.com/hgrecco/ca-migration.

## Author Contributions

MC, CP-C, and HG designed the experiments, discussed the model, and wrote the paper. MC and LC performed the experiments and acquired the data. MC and HG performed the data analysis, implemented the computational code, and run the simulations. All authors reviewed and edited the paper.

## Conflict of Interest

The authors declare that the research was conducted in the absence of any commercial or financial relationships that could be construed as a potential conflict of interest.

## Supplementary Material

Supplementary Video 1 Smaller spheroids under migration assay show high cell movement away from colony.

Supplementary Video 2 Larger spheroids remain clustered and few cells detached during migration assay.

Supplementary Video 3 Some cells show mechanical interactions and migrate collectively, attached to each other.

Supplementary Video 4 Some cells change their migration direction, re-clustering after several hours of migration.
