# Supplementary material for: An Integrative and Modular Framework to Recapitulate Emergent Behavior in Cell Migration
Source: Front Cell Dev Biol. 2020 Dec 22;8:615759. doi: 10.3389/fcell.2020.615759 (PMC7783155; doi:10.3389/fcell.2020.615759)
Supplement: Supplementary file 5 [file Data_Sheet_1.PDF]

Supplementary Material

1 SUPPLEMENTARY DATA

1.1 Figures

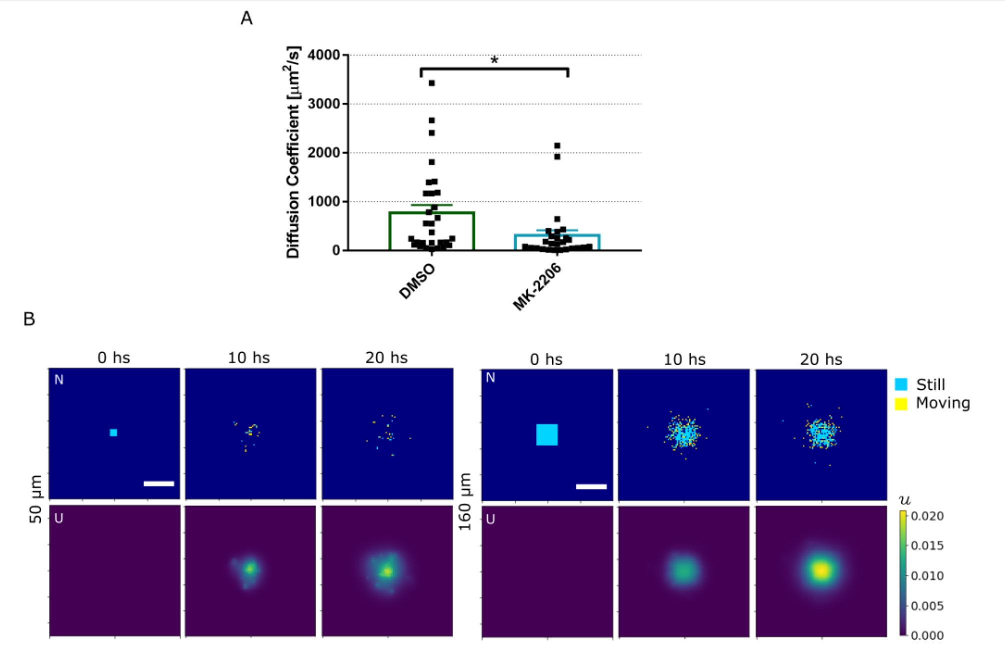

Figure S1. A. Quantification of diffusion coefficient of cells in monolayer with vehicle DMSO and treated with 7 μM MK-2206 (mean $\pm$ SEM, * p-value $<$ 0.05). B. Matrices of cell and chemical distribution (N and U) at times 0, 10 and 20 hs for a small 50 μm (top) and big 160 μm (bottom) geometry for $r = 0.4$. Cells in yellow moved in the last iteration while cells in blue remained still. Scale bar = 200 μ m.

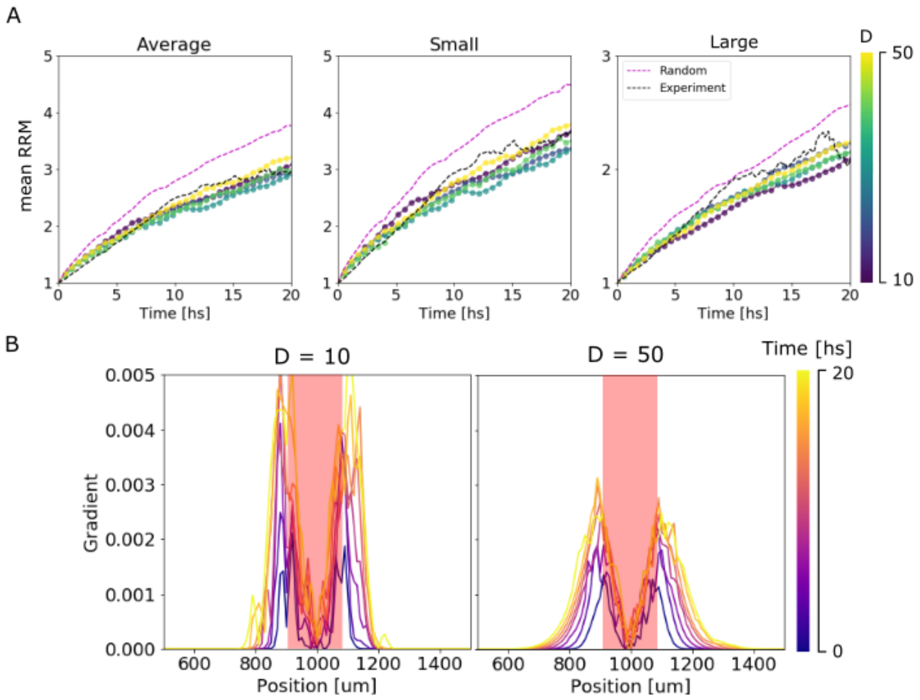

Figure S2. A. Range of diffusion coefficients valid for the simulations did not show great differences for the average and small subset of spheroids. In large spheroids higher diffusion coefficients seems to correlate with more migration. B. Chemical gradient in the horizontal direction for different values of D show, as expected, higher values for lower D due to accumulation of chemo-attractant. In red, initial spheroid diameter is indicated.

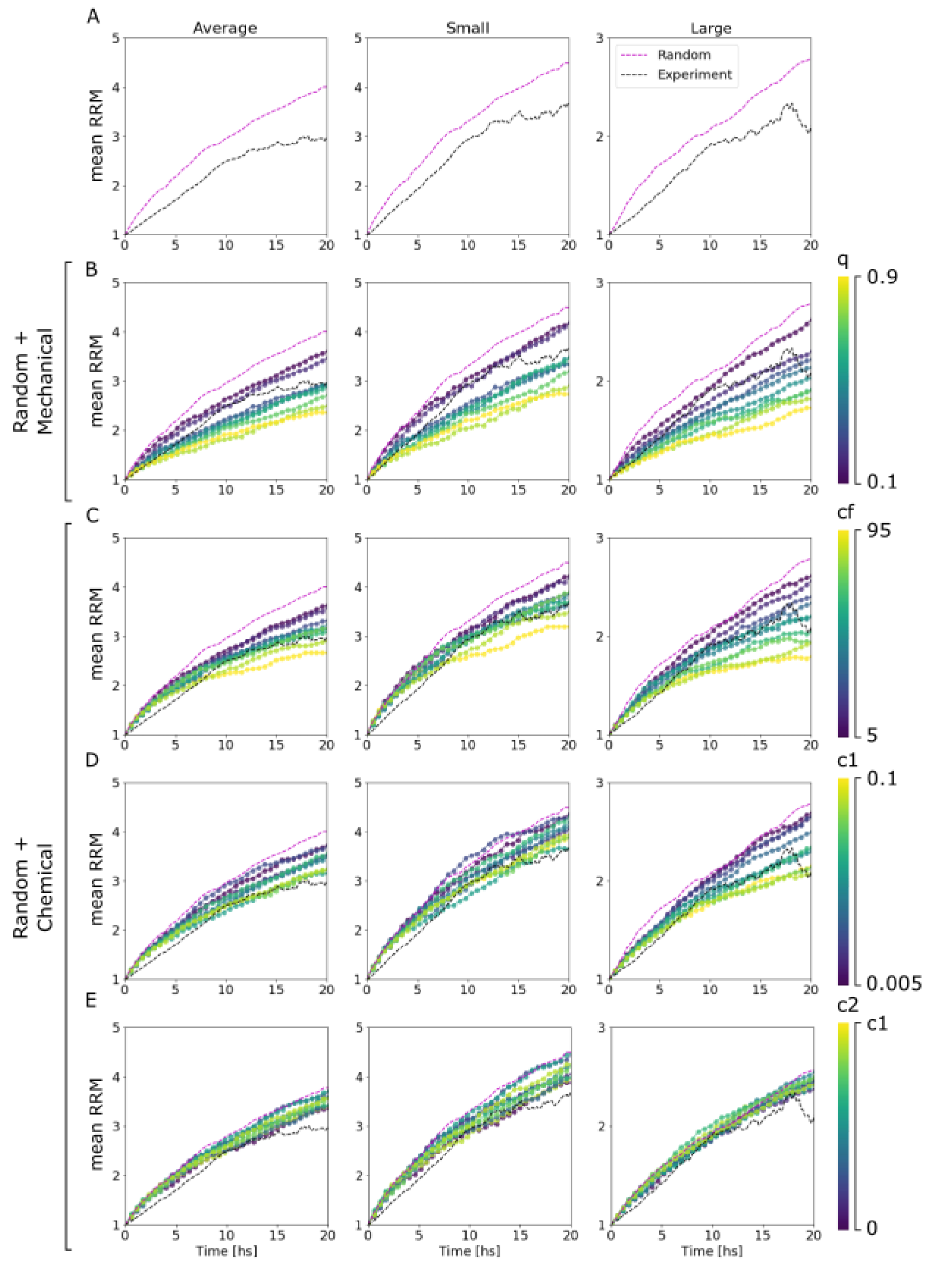

Figure S3. A. Dynamic range of simulations. B. q range for different subsets of spheroids. C, D, E range explored for c_f, c_1 and c_2 for the different subsets of spheroids.
